# A 46-year-old Chinese woman presenting with retroperitoneal follicular dendritic cell sarcoma: a case report

**DOI:** 10.1186/1752-1947-8-113

**Published:** 2014-04-03

**Authors:** Taize Yuan, Qiuxiang Yang, Huanhuan Zhang, Jian Li, Xiuping Zhang

**Affiliations:** 1 Department of Radiation Oncology, Affiliated Tumor Hospital, Guangzhou Medical University, No.78 Hengzhigang, Road, Guangzhou, Guangdong Province 510095, China

**Keywords:** Follicular dendritic cell sarcoma, Immunohistochemistry, Pathology, Retroperitoneal, Therapy

## Abstract

**Introduction:**

Follicular dendritic cells are non-phagocytic, non-lymphoid cells of the immune system that are necessary for antigen presentation and the regulation of reactions in the germinal centers of the lymph nodes. Follicular dendritic cell sarcoma is an unusual cancer, particularly in the intra-abdominal region. In the present report we describe an unusual case of retroperitoneal follicular dendritic cell sarcoma that emphasizes the difficulty of diagnosing and treating this tumor. Retroperitoneal follicular dendritic cell sarcoma has only been rarely reported in the literature to date.

**Case presentation:**

A 46-year-old Chinese woman of Han ethnicity presented with chronic right lower quadrant abdominal pain over the preceding 4 weeks. The tumor was resected and submitted to histopathological examination. The case was verified as retroperitoneal follicular dendritic cell sarcoma by microscopic examination and immunohistochemical analysis. After diagnosis, she received postoperative radiotherapy and chemotherapy. She has survived 3 years postoperatively, although she has a pulmonary metastasis.

**Conclusions:**

Retroperitoneal follicular dendritic cell sarcoma may demonstrate aggressive potential. This study indicated that postoperative adjuvant radiotherapy and chemotherapy could extend the survival of a patient with retroperitoneal follicular dendritic cell sarcoma.

## Introduction

Follicular dendritic cells (FDCs) are among the accessory cells of the lymphoid system. Their main function is to trap and present antigens and immune complexes to B cells. FDCs are found mainly in the germinal centers of the lymphoid tissue, where they form a tight meshwork. FDCs feature complement receptors and human leukocyte antigen-DR on their surfaces [1,2]. Lymphoid follicles are found in the lymph nodes and in extranodal lymphoid tissue [3]. Follicular dendritic cell sarcoma (FDCS), which was first reported by Monda *et al.*[1] in 1986, is a rare neoplastic proliferation that exhibits the morphological and immunophenotypic features of FDCS. To date, approximately 106 cases of FDCS have been reported in the literature, of which 46 occurred intra-abdominally [4]. We report a case with retroperitoneal FDCS that emphasizes the difficulty of diagnosing and treating this tumor.

## Case presentation

**Figure 1:**
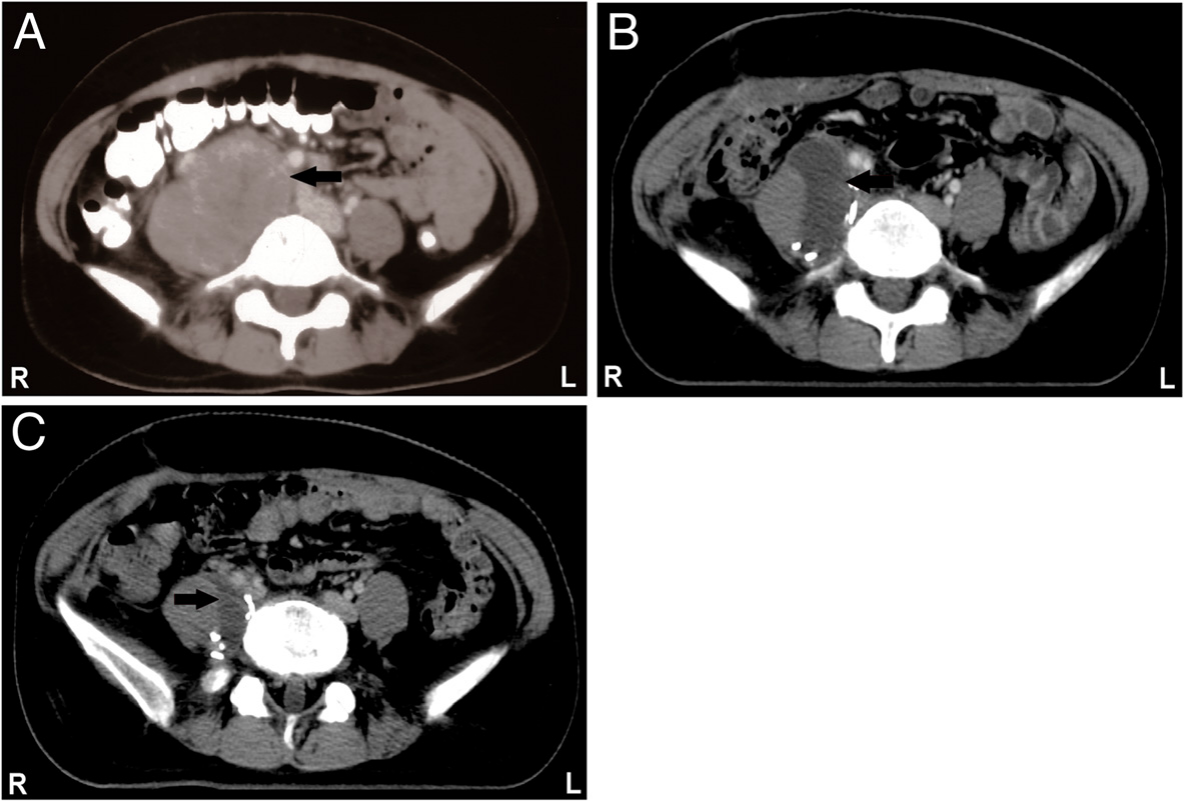
**Computed tomography images of retroperitoneal follicular dendritic cell sarcoma. A**: Contrast-enhanced computed tomography scan of the abdomen revealing an 8.6cm retroperitoneal mass (arrow); **B**: liquid dark area (arrow) observed in the surgical area after operation; **C**: significant shrinking of the liquid dark area (arrow) was observed after adjuvant therapy.

A 46-year-old Chinese woman of Han ethnicity presented with a 4-week history of chronic right lower quadrant abdominal pain without other associated symptoms. A general systemic examination was normal except for a large mass in her right lower quadrant. There was no significant past medical history. Routine biochemical and hematological tests were within normal limits. A chest X-ray also revealed no abnormalities. A subsequent abdominal computed tomography (CT) scan revealed a 8.6×6.0cm oval-shaped mass, with a clear boundary and mild-moderate enhancement, in her lower right retroperitoneal region, pressing against her right psoas muscle and her right iliac artery (Figure 1A).

She underwent an operation to resect the mass. Surgical exploration revealed a retroperitoneal mass measuring 9×6cm adjacent to her transverse colon, her right psoas muscle and her vertebral column and covering her inferior vena cava. No associated lymphadenopathy was observed. Because the mass had infiltrated her right psoas muscle and was attached to her right iliac artery and her inferior vena cava, most of the gross tumor was resected, and the margin was positive. A CT scan revealed a liquid dark area in the surgical area after the operation (Figure 1B).

**Figure 2:**
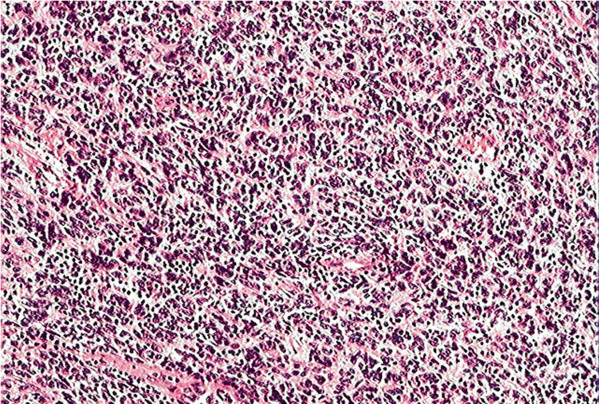
**Microscopic appearance of the retroperitoneal follicular dendritic cell sarcoma.** The histopathological appearance indicates that the tumor is composed of spindle cells arranged in a storiform pattern and that these spindle cells are admixed with lymphocytes (hematoxylin and eosin stain, ×100).

**Figure 3:**
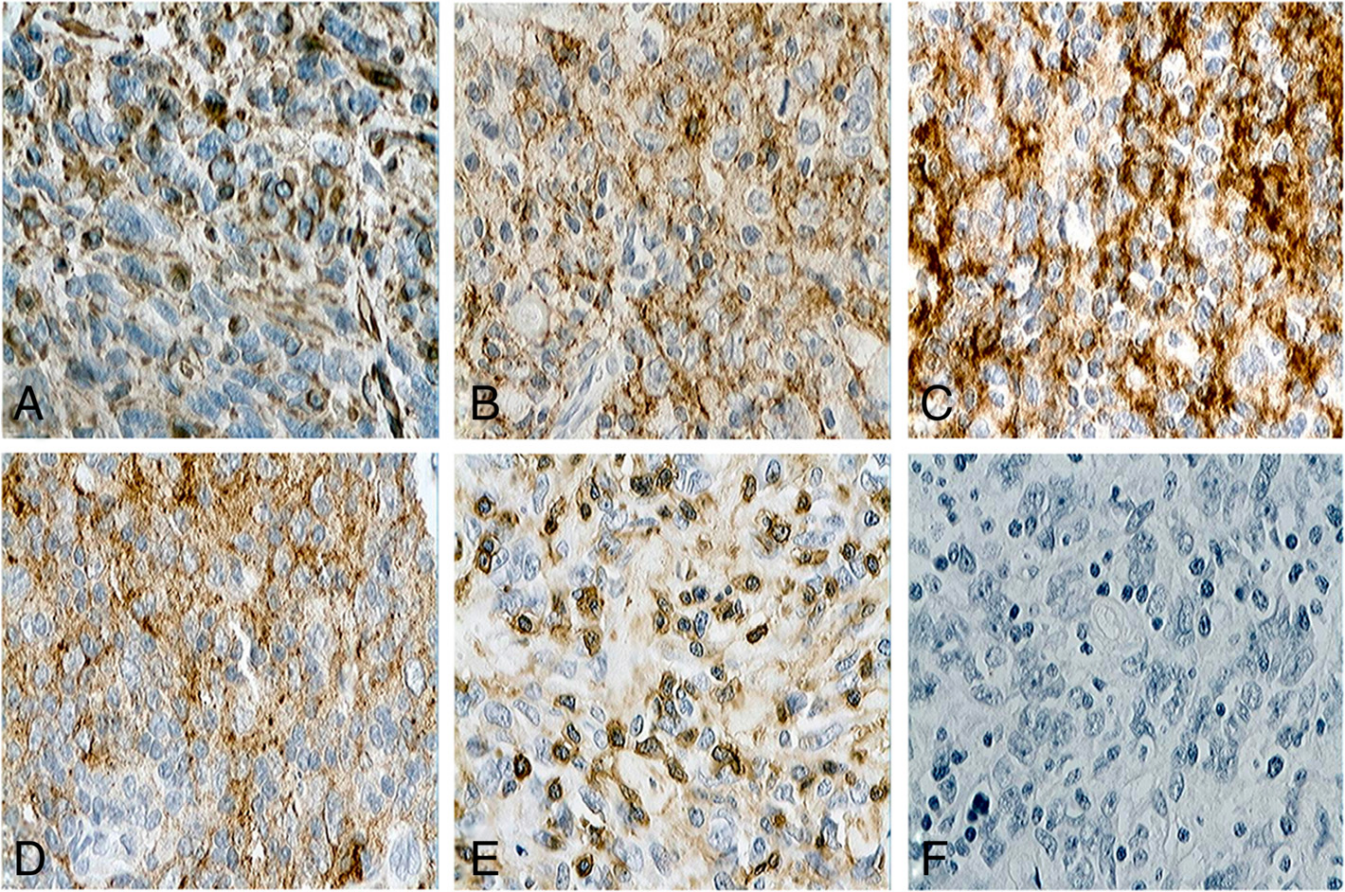
**Immunohistochemical features of retroperitoneal follicular dendritic cell sarcoma (paraffin immunohistochemical stain×400). A**: Positive immunohistochemical staining for vimentin; **B**: Positive immunohistochemical staining for CD21; **C**: Positive immunohistochemical staining for CD35; **D**: Positive immunohistochemical staining for D2-40; **E**: Positive immunohistochemical staining for leukocyte common antigen; **F**: Negative immunohistochemical staining for cytokeratin.

A cut section revealed a homogenous solid mass with a rough surface. Multiple sections were taken from various areas of the tumor. Microscopic examination revealed that the tumor was composed of fat to long spindle cells with multinodular infiltration that were arranged in a storiform pattern (Figure 2). The tumor exhibited necrosis, cell atypia and obvious mitosis. The mitotic events numbered up to 12 per 10 high-powered fields, and there was focally marked nuclear atypia, including pleomorphic, multinucleated cells. Furthermore, the tumor cells exhibited positive immunohistochemical staining for vimentin, CD21, CD35, D2-40 and leukocyte common antigen (Figure 3A to 3E) as well as negative staining for cytokeratin (Figure 3F), CD20, CD30, CD117, epithelial membrane antigen and S-l00 (not shown). Based on these histopathological and immunohistochemical findings, the patient was diagnosed with FDCS of the retroperitoneal lymph node.

She received postoperative sequential chemotherapy and radiotherapy. Three cycles of chemotherapy with a standard dose of cyclophosphamide, doxorubicin, vincristine and prednisone (CHOP) were initiated in July 2010. A total radiation dose of 60Gy was then administered over 6 weeks, followed by another three cycles of chemotherapy with CHOP. To evaluate the efficacy of the adjuvant therapy, an abdominal CT scan was performed, which revealed that the liquid dark area had shrunk significantly (Figure 1C).

Hospital records indicate that the patient has reached 3 years of follow-up. Although she is alive, she has a right pulmonary metastasis.

## Discussion

FDCS is a rare tumor that originates in the germinal centers of the lymph nodes in FDCS and has low malignant potential. Monda *et al.*[1] first described the malignant neoplasm in 1986. FDCS occurs mainly in adults; the average age is 47 years (14 to 77 years). The incidence rate is the same for men and women [5]. The most common clinical symptom is a painless and slow-growing lymph node. FDCS is found mostly in the lymph nodes, particularly the cervical lymph nodes and occasionally in the axillary and mediastinal lymph nodes. FDCS is also occasionally found outside the lymph nodes. Extranodal FDCS has been found in the tonsils, nasopharynx, liver, spleen and gastrointestinal tract [3,6-9].

Pathological diagnosis is challenging [10] and may require a combination of morphological, immunophenotypical, cytochemical and electronmicroscopic analyses [2]. FDCS may be suspected if the tumor exhibits distinct microscopic features, such as a storiform arrangement of spindle-shaped cells, indistinct cell borders and a background of lymphocytes scattered throughout the neoplastic cells [11]. The morphology of our case is typical of FDCS, although various studies have reported atypical presentations in the form of a multifocal disease, increased mitosis, giant cell transformation and wide areas of necrosis. To confirm the tumor, immunohistochemical staining is required to detect the most widely used FDC markers, including CD21, CD35, vimentin and D2-40, which can distinguish FDCS from other spindle cell neoplasms [11,12]. S-100 and certain vascular or muscular markers may also help to distinguish this tumor from other tumors, such as malignant peripheral nerve sheath tumors and gastrointestinal stromal tumors [13].

A complete surgical excision is the appropriate initial therapy [3]. Adjuvant radiotherapy or chemotherapy appears to be indicated in cases of incompletely resected lesions or adverse pathological features during recurrence [14]. However, because of the rarity of the lesion and the retrospective nature of the published literature, the role of adjuvant therapy (radiotherapy or chemotherapy) remains unclear. A few recent reports have suggested that FDCS should be viewed as an intermediate-grade malignancy that presents a substantial risk of metastasis to the lung, liver, intra-abdominal soft tissues and lymph nodes [11,15].

## Conclusions

As in this patient, retroperitoneal FDCS may demonstrate aggressive potential. This study indicated that postoperative adjuvant radiotherapy and chemotherapy could extend the survival of a patient with retroperitoneal FDCS.

## Consent

Written informed consent was obtained from the patient for publication of this case report and accompanying images. A copy of the written consent is available for review by the Editor-in-Chief of this journal.

## Abbreviations

CHOP: Cyclophosphamide, doxorubicin, vincristine and prednisone; CT: Computed tomography; FDCs: Follicular dendritic cells; FDCS: Follicular dendritic cell sarcoma.

## Competing interests

The authors declare that they have no competing interests.

## Authors’ contributions

TY and QY contributed equally to this work; TY, QY and XZ analyzed and interpreted the data from our patient and drafted the manuscript. HZ and JL performed the histological examination of the tumor. All authors read and approved the final manuscript.
